# Organelle Dynamics in Apicomplexan Parasites

**DOI:** 10.1128/mBio.01409-21

**Published:** 2021-08-24

**Authors:** Julie M. J. Verhoef, Markus Meissner, Taco W. A. Kooij

**Affiliations:** a Department of Medical Microbiology, Radboudumc Center for Infectious Diseases, Radboud Institute for Molecular Life Sciences, Radboud University Medical Center, Nijmegen, Netherlands; b Faculty of Veterinary Medicine, Experimental Parasitology, Ludwig Maximilian University, Munich, Germany; Albert Einstein College of Medicine

**Keywords:** *Plasmodium*, *Toxoplasma*, organelle segregation, mitochondrion, dynamins

## Abstract

Apicomplexan parasites, such as Toxoplasma gondii and Plasmodium falciparum, are the cause of many important human and animal diseases. While T. gondii tachyzoites replicate through endodyogeny, during which two daughter cells are formed within the parental cell, P. falciparum replicates through schizogony, where up to 32 parasites are formed in a single infected red blood cell and even thousands of daughter cells during mosquito- or liver-stage development. These processes require a tightly orchestrated division and distribution over the daughter parasites of one-per-cell organelles such as the mitochondrion and apicoplast. Although proper organelle segregation is highly essential, the molecular mechanism and the key proteins involved remain largely unknown. In this review, we describe organelle dynamics during cell division in T. gondii and P. falciparum, summarize the current understanding of the molecular mechanisms underlying organelle fission in these parasites, and introduce candidate fission proteins.

## INTRODUCTION

Members of the phylum Apicomplexa are single-cell, intracellular parasites that can cause many important human and animal diseases, including malaria, toxoplasmosis, and cryptosporidiosis, affecting millions of people every year. These unique eukaryotes have a fascinating biology, which enables them to grow and thrive within other eukaryotes and clearly distinguishes them from other pathogens such as viruses and bacteria. Apicomplexan parasites have complex life cycles, which in large part constitute an obligate intracellular replication cycle. In many cases, this often-rapid increase of parasite numbers goes hand in hand with inflammation and tissue damage and is a result of a short replication cycle and very efficient cell division. During the 48-h intraerythrocytic replication cycle of Plasmodium falciparum, a causative agent of malaria, one parasite can generate up to 32 merozoites, each capable of invading another red blood cell. Moreover, liver-stage P. falciparum can generate up to 40,000 merozoites from a single sporozoite in 7 days, highlighting the extremely fast replication capability of these parasites (1). In contrast to the familiar binary division of mammalian, plant, fungal, and bacterial cells, apicomplexan parasites replicate by *de novo* assembly of daughter cells within the parental cell. Depending on the number of newly formed parasites and the timing of nuclear division, this process is called schizogony, endodyogeny, or endopolygeny (2).

Both intracellular replication of P. falciparum within host erythrocytes and hepatocytes and extracellular replication of the oocyst in the mosquito vector happen via schizogony. During schizogony, asynchronous nuclear division results in a nongeometric expansion, after which a final round of nuclear division leads to the coordinated segmentation of daughter cells (2). Although it was previously thought that this last round of nuclear division happens in a synchronous manner, a recent study from Rudlaff et al. demonstrated that this happens asynchronously (3). Toxoplasma gondii, the causative agent of toxoplasmosis, replicates via endodyogeny during the tachyzoite stage. During this process, DNA replication is immediately followed by the assembly of two daughter cells within the parental parasite (2). Endopolygeny is a mode of replication that is used by parasites such as Sarcocystis neurona. These parasites undergo multiple rounds of mitosis without nuclear division, resulting in a polyploid nucleus. Only during daughter cell assembly, the last round of mitosis is followed by nuclear division and the packaging of haploid nuclei in the daughter parasites (2, 4). During these processes, parasites need to have extensive spatial and temporal control to ensure proper segregation of organelles and distribution of genetic material over daughter cells.

Like almost all eukaryotes, apicomplexan parasites contain a nucleus, an endoplasmic reticulum (ER), and a Golgi complex. However, they also harbor a specialized set of secretory organelles, including the rhoptries, micronemes, and dense granules, which are important for parasite invasion and establishment of parasitophorous vacuole (PV). These organelles are formed *de novo* during late intracellular stages (5). Additionally, Apicomplexa possess an inner membrane complex (IMC) located directly beneath the plasma membrane. The IMC consists of flattened membrane sacs called alveoli and plays an important role in parasite replication, motility, and host cell invasion (6). Furthermore, Apicomplexa harbor two singular organelles of endosymbiotic origin, the mitochondrion and a plastid organelle called the apicoplast, which both have their own reduced genomes (7). The apicomplexan mitochondrion differs greatly from the host mitochondria on a molecular and functional level (8). One striking difference is that P. falciparum asexual blood stages use their electron transport chain primarily for pyrimidine biosynthesis, rather than ATP synthesis, manifesting in the loss of cristae (9, 10). The apicoplast was acquired by secondary endosymbiosis of a red alga but has lost its photosynthetic capacity (11, 12). This organelle is characterized by four membranes and plays a key role in major metabolic pathways, such as generation of isoprenoid, fatty acids, and heme (13). The essentiality of the mitochondrion and apicoplast in apicomplexan parasites is demonstrated by the fact that these organelles are well-established drug targets (14, 15). A recent subcellular atlas of the *Toxoplasma* proteome confirmed a significant overrepresentation of essential functions in these endosymbiotic organelles (16, 17).

As each individual parasite harbors only a single mitochondrion and apicoplast, it is highly important that they are properly divided and distributed over daughter cells during cell division. Unlike organelle fission in mammalian, yeast, and plant cells, almost nothing is known about organelle fission in apicomplexan parasites. Other eukaryotic cells often harbor multiple mitochondria that are able to rapidly change in size, shape, and position. They undergo continuous fission and fusion events to adapt to energy needs of the cell (18). In contrast, organelle division in apicomplexan parasites is tightly linked to cell division, and spontaneous fusion or fission events have not been observed (5, 19, 20). Although loop formation of the mitochondrion in T. gondii and P. falciparum could suggest the presence of self-fusion events, no components of a fusion machinery have been identified, indicating that fusion of the individual organelles is redundant in these parasites. (19, 20). In this review, we will describe organelle dynamics during cell division of the most commonly studied apicomplexan parasites, P. falciparum and T. gondii, with a focus on the apicoplast and mitochondrion. Furthermore, we will summarize the current understanding of the molecular mechanisms underlying organelle fission in these parasites and introduce candidate fission proteins. Finally, we propose possible fission scenarios during schizogony and speculate about future directions to unravel these essential processes.

## ORGANELLE DYNAMICS DURING CELL DIVISION

During eukaryotic cell division, the cytoskeleton, membranes, and organelles change dramatically. In order to be fully functional, each daughter cell must be equipped with a complete set of organelles. A dividing cell is faced with the challenge of partitioning many different organelles that can vary in size, per-cell number, shape, and location. In mammalian cell division, larger and more complex organelles, such as the ER, Golgi, and nuclear envelope, must be extensively remodeled and disassembled before being distributed and reformed (18). Smaller organelles that occur in larger numbers per cell, such as mitochondria, are fragmented prior to their distribution during cell division (21). Unlike mammalian and plant cells, apicomplexan parasites harbor only one of each of their endosymbiotic organelles that demonstrate highly dynamic structures during the replication cycle. Organelles linked to the endomembrane system, such as ER, Golgi, and secretory organelles, are distributed over daughter cells using a combination of *de novo* synthesis and recycling. However, endosymbiotic organelles need to replicate their genomes and undergo division, similarly to their bacterial ancestors. Organelle division is tightly coupled to cell division and happens in a highly organized and consecutive manner.

### Structural changes of organelles during Toxoplasma gondii replication.

**FIG 1:**
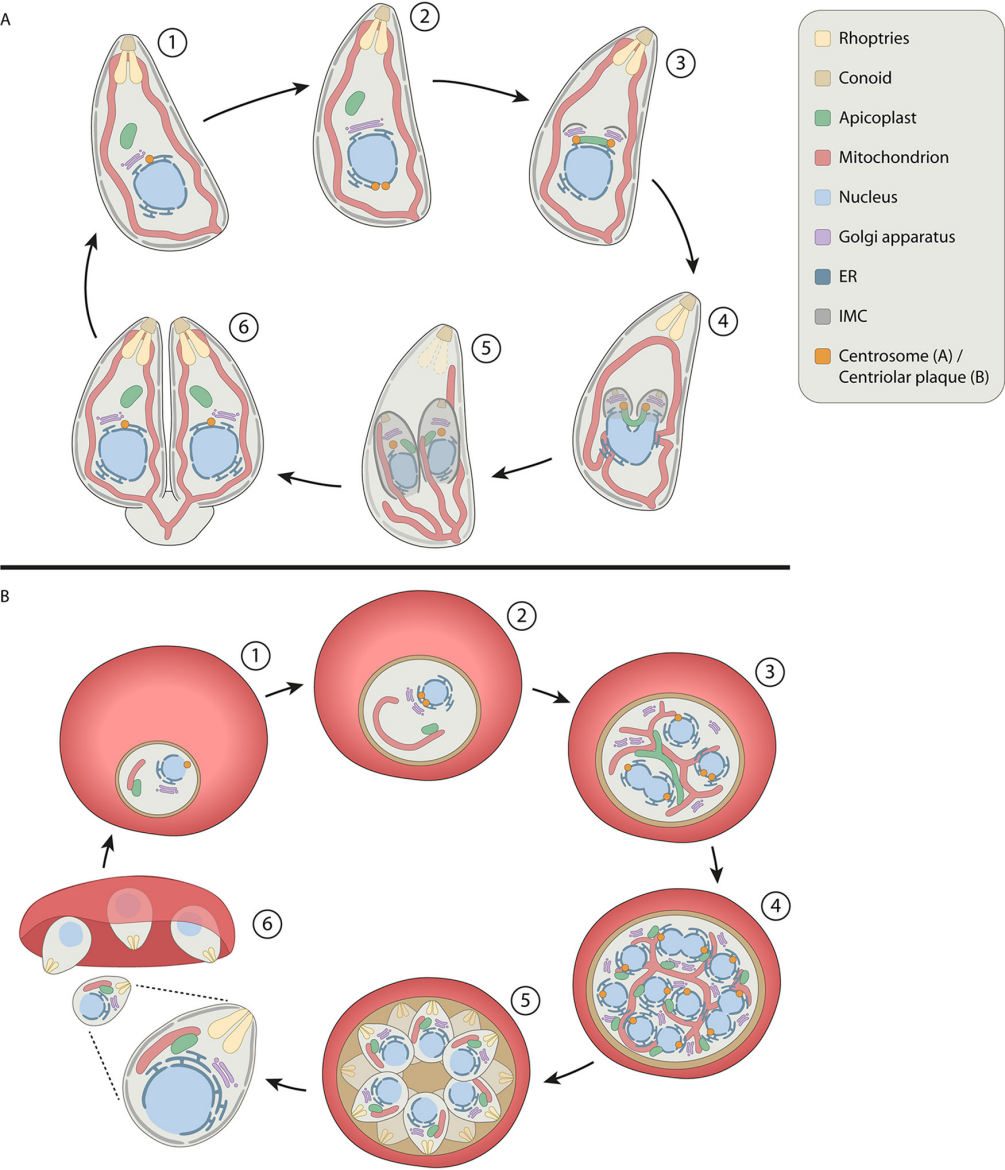
Schematic overview of organelle morphology during endodyogeny in T. gondii and schizogony in P. falciparum. (A) Replication cycle of T. gondii tachyzoites. (1) Mature parasite. (2) Lateral elongation of the Golgi and migration and duplication of the centrosome at the basal site of the nucleus. (3) Centrosomes migrate back to the apical side of the nucleus and associate with the Golgi, which undergoes medial fission. The apicoplast also associates with the centrosomes and undergoes lateral extension. Budding is initiated with the formation of the IMC of daughter parasites. (4) Further formation of the IMC scaffold. Apicoplast remains associated with the centrosomes resulting in a U-shape. Nucleus and surrounding ER start to divide and enter the daughter parasites. (5) Fission of the apicoplast and nucleus with the ER. IMC scaffold encapsulates divided organelles. Extensions of the mitochondrion enter the daughter parasites. Degradation of parental secretory organelles and IMC. (6) Daughter parasites emerge, formation of the secretory organelles, establishment of the mitochondrial lasso, formation of the basal body. Only at the very last moment of division, mitochondria are separated at the basal end. (B) Asexual replication of P. falciparum in red blood cells. (1) Ring-stage parasite. (2) Elongation of the mitochondrion and division of the centriolar plaque (CP) and Golgi. ER forms extensions into the cytosol. (3) Further elongation and branching of the mitochondrion and apicoplast. Further replication of the Golgi and CP. Replication and expansion of the ER surrounding the dividing nuclei. (4) Apicoplast divides and associates with mitochondrial branches. Last round of nuclear division. (5) Mitochondrial division and formation of the daughter parasites. (6) Egress of merozoites from the red blood cell.

During endodyogeny in T. gondii, individual organelles are divided and distributed equally in assembling daughter parasites in a tightly synchronized manner. The division process starts with fission of the Golgi and migration of the centrosome from the apical to the basal side of the nuclear envelope (Fig. 1A). After duplication, the centrosomes return to the apical side of the nucleus (5, 22). The importance of this migration is not yet understood. At the same time, the single Golgi apparatus of the parasite undergoes lateral elongation and medial fission (5, 22, 23). After Golgi fission, centrosomes localize at the inner ends of the divided Golgi. The apicoplast also associates with the centrosomes and undergoes lateral extension (5, 24). The scaffold of the two daughter cells, consisting of the conoid, IMC, and subpellicular microtubules, starts to form and encapsulate the Golgi. The opposite ends of the elongated apicoplast are drawn into the growing daughter cells. The organelle remains associated with the centrosomes, resulting in a U-shaped structure (24, 25). Next, the apicoplast undergoes medial fission and both daughter apicoplasts are packed in the assembling daughter cells. The mitochondrion typically has a lasso-shaped structure associating with the periphery of the parasite (19, 26). During G_1_ and apicoplast elongation stages, the apicoplast and mitochondrion transiently associate with each other (5). At the start of daughter IMC formation, the mitochondrion starts to form branches at multiple locations along its length. The ER forms a network-like structure with extensions from the nuclear envelope (5, 27). As the daughter scaffold elongates, DNA replication is completed and the nucleus lobulates. Following nuclear division, the ER enters the forming daughter scaffolds from the basal side together with the nucleus. Remarkably, the mitochondrion is completely excluded from the developing daughter cells until very late during cell division. Once initiated, the entry of the mitochondrial extensions into the daughter cells is very fast. Here, the lasso-shaped form of the mitochondrion is reestablished, but the newly formed mitochondria remain attached at the basal part for an unknown period of time (5, 28). Ultimately, daughter mitochondria are separated at the basal part. Finally, maternal organelles and structures, such as micronemes, rhoptries, the IMC, and plasma membrane, are almost quantitatively recycled from the parental to the daughter parasites (29). This recycling process depends on a highly dynamic F-actin network that organizes the residual body and connects individual parasites to ensure equal distribution of maternal organelles to the forming daughter cells (29, 30). Indeed, while earlier studies suggested a relatively minor role of the parasites’ actomyosin system during replication and focused on its role in gliding motility and host cell invasion, recent findings demonstrate that actin and unconventional myosins, such as MyoF, play crucial roles in organelle recycling, apicoplast segregation, and organization of the parasites’ endomembrane system (30–33).

### Structural changes of organelles during P. falciparum replication.

In contrast to the relatively straightforward cell division of T. gondii where the parental cell segments into two daughter cells, the process of schizogony in P. falciparum is more complex. During erythrocytic schizogony of P. falciparum, up to 32 daughter parasites can be formed in a single parental cell. Consequently, organelles undergo drastic morphological changes and complicated fission patterns (Fig. 1B). In early ring-stage parasites, the ER has a simple crescent shape around the nucleus (20). The single Golgi apparatus of the parasite localizes closely to the ER and the nucleus (34). The apicoplast has a rounded shape, while the mitochondrion is typically slightly elongated and has a tubular form (20). As the parasite develops into a trophozoite, the ER forms extensions into the cytosol and around the food vacuole. The mitochondrion elongates further through the cytoplasm and starts to form branches, while the apicoplast mostly retains its rounded shape. During these earlier stages of parasite development, the apicoplast and mitochondrion often localize in close proximity to each other. In contrast to T. gondii parasites, P. falciparum parasites lack canonical centrosomes. They organize their mitotic spindle from a centriolar plaque, which is embedded in the nuclear envelope (35). The centriolar plaque duplicates and migrates to opposite sides of the nucleus prior to nuclear division (36). This pattern repeats itself coincident with the asynchronous nuclear division. Similar to that in T. gondii, the Golgi apparatus also duplicates prior to nuclear division. Only after the onset of nuclear division in early schizonts, the apicoplast elongates and the mitochondrion starts to form a more complex branched structure (20, 37). The ER forms a highly branched mesh-like network, and further multiplication of the Golgi occurs (34, 38). As the apicoplast branches out, the number of contact points with the mitochondrion increases. The apicoplast divides during late-stage schizogony prior to mitochondrial division (3). Daughter apicoplasts associate with the smaller branches of the mitochondrion. The mitochondrion only divides very late during schizogony and segregates as a pair with the apicoplast into the new daughter merozoites. How and when the ER is divided and distributed during schizogony remains largely unexplored. In newly formed merozoites, the ER has again a crescent-like shape around the nucleus, while the apicoplast is rounded and the mitochondrion has a slightly elongated tubular structure (20).

Interestingly, similar apicoplast and mitochondrial fission patterns have been observed in liver-stage parasites but on a much larger scale, with simultaneous formation of tens of thousands of merozoites (39). During the extremely fast rounds of nuclear division in liver-stage parasites, the apicoplast and mitochondrion become extensively elongated and branched structures. The apicoplast divides with surprising synchronicity along its length while remaining closely associated with the mitochondrion, which forms fingerlike structures. Similar to intraerythrocytic schizogony, during hepatic schizogony the apicoplast always divides prior to the mitochondrion. Shortly before formation of the daughter parasites, the mitochondrion divides in a similarly synchronous manner.

### Organelle contact sites.

During the cell division process of both T. gondii and P. falciparum, there are several moments of membrane contact between different organelles. Interestingly, in human cells, association between the ER and mitochondrion is needed for the initial step of mitochondrial division (40). The ER tubules wrap around the mitochondria and facilitate actin-myosin-mediated mitochondrial constriction. This preconstriction step is required to decrease the mitochondrial diameter by approximately half, allowing the mitochondrial division machinery to be recruited. Recently, these mitochondrion-ER contact sites have also been implicated in phospholipid and calcium transfer during division, suggesting that these contact sites also present a signaling platform for metabolite exchange that facilitates membrane remodeling and division (41, 42). Although membrane contact points between the mitochondrion and ER in T. gondii and P. falciparum have not been reported, close association between the apicoplast and extensions of the ER has been observed in these parasites (43–45). Association of other four-membrane-bound plastids with the ER has also been observed in heterokont, haptophyte, and cryptomonad algae (46). In plants, contact sites between the chloroplast and the ER are indicated to be involved in lipid transport (47). Apicoplast-ER contact sites in apicomplexan parasites might also play a role in lipid distribution, which is needed for membrane remodeling and organelle dynamics (43). So far there is no evidence that ER tubules physically wrap around the apicoplast to mediate apicoplast constriction; however, this remains largely unexplored.

The apicoplast and mitochondrion in T. gondii and P. falciparum divide subsequently while they have several transient contact sites. These contact sites have been observed with fluorescent and electron microscopy during both intraerythrocytic and hepatic schizogony (20, 39, 44, 48). It has been suggested that organelle contact facilitates metabolic exchanges important for the biosynthesis pathway of heme, isoprenoid, iron-sulfur clusters, and fatty acids (44, 48–53). The contact points between these organelles might also represent a mechanism to ensure that every daughter parasite receives only one of each organelle (20, 39). Since apicoplast and mitochondrial fission happen in two subsequent steps, it is also possible that these contact sites allow exchange of a putatively shared fission machinery involved in the division of both organelles. However, so far this theory remains unexplored.

## MECHANISMS OF ENDOSYMBIOTIC ORGANELLE SEGREGATION

### Distribution of endosymbiotic organelles.

While the unusual morphology of apicomplexan parasites suggests the presence of rather unique machineries and proteins, other aspects of organelle division and distribution during the formation of daughter cells appear to be a common theme throughout the eukaryotic kingdom. Thus, distribution of divided organelles in eukaryotic cells typically involves coordinated remodeling of actin and the microtubule cytoskeleton. Microtubules and microtubular dynamics are critical for daughter cell assembly (31). Interestingly, cell division is coordinated by a homolog of the striated rootlet fiber of algal flagella, striated fiber assemblins that are expressed only during division and connect the centrosome with the microtubule organization centers of the developing daughter cells, thereby defining the symmetry axis for division. Furthermore, centrosomes are tightly linked with the apicoplast, which is thought to be required for proper segregation during cytokinesis and allows to distribute apicoplasts evenly among daughter parasites (24). In contrast, until recently the multiple roles of the parasites’ actomyosin system during replication remained obscure, since the organization and functions of the actin cytoskeleton in apicomplexan parasites remained elusive due to the lack of adequate reagents to visualize F-actin and *in vitro* data that suggested that only short filaments can be formed in an unusual, isodesmic polymerization mechanism (54). However, recent studies clarified that apicomplexan actin is well capable of forming long filaments of up to 30 μm in a cooperative polymerization mechanism, as seen for canonical actins (55, 56). Furthermore, with the application of the actin chromobody it was possible to visualize F-actin in T. gondii and P. falciparum and to explain surprising effects caused by disruption of F-actin dynamics or parasite myosins (30, 33, 57–60).

As in other eukaryotes, parasite actin plays crucial roles during parasite division and is involved in recycling of maternal organelles as well as apicoplast inheritance. Interestingly, the unconventional myosin F (MyoF) appears to be the central motor protein for these diverse functions. Ablation of *Tg*MyoF leads to loss of the apicoplast and affects the dynamics, positioning, and movement of organelles of the endomembrane system (33, 59, 61). To date, the exact function of MyoF and actin during apicoplast segregation is unknown. Interestingly, depletion of the actin nucleator Formin-2, which is localized close to the apicoplast, leads to a similar defect in apicoplast segregation in both T. gondii and P. falciparum (57). Importantly, the actomyosin system appears to act downstream of apicoplast fission, since individual parasites can possess several apicoplasts, while others do not obtain a single apicoplast, upon interference with the actomyosin system.

While the role of actin, striated fiber assemblins, and microtubules in division and segregation of the apicoplast is well documented, their role in mitochondrial segregation is still obscure and requires further analysis.

### Ancestral and eukaryotic division machinery.

**FIG 2:**
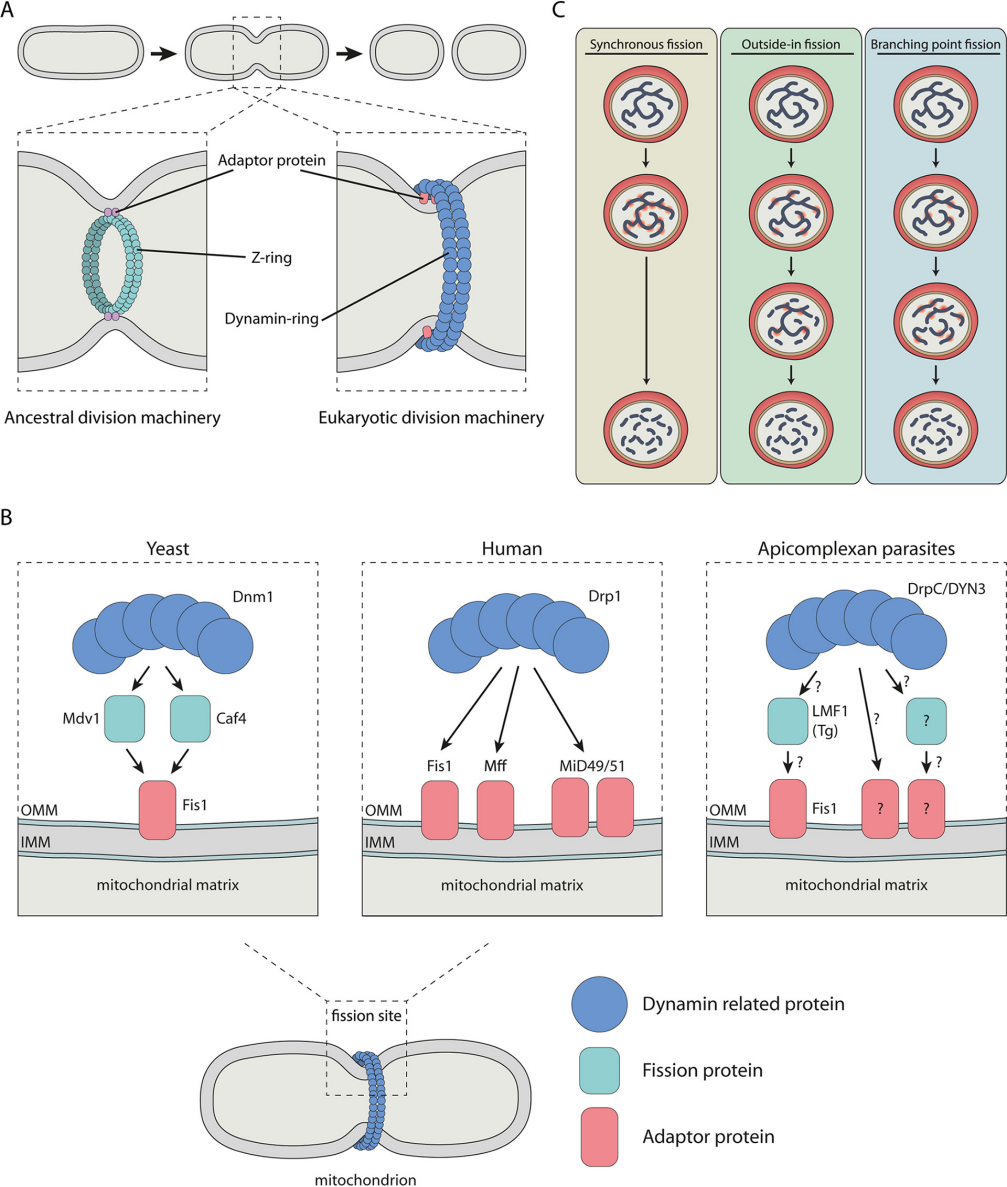
Schematic representations of organelle division mechanisms. (A) Endosymbiotic organelle division machineries. Endosymbiotic organelles are divided by the ancestral FtsZ-based division machinery where the Z-ring forms beneath the inner organelle membrane and/or the eukaryotic dynamin-based division machinery in which the dynamin ring forms at the cytosolic side of the outer organelle membrane. (B) Adaptor proteins recruit the mitochondrial division machinery in yeast, human, and apicomplexan parasites. In yeast, the membrane-anchored Fis1 recruits adaptor proteins Mdv1 and Caf4, which in turn recruit Dnm1 to form the constrictive ring. In human cells, multiple membrane-anchored adaptor proteins, including Fis1, Mff, and MiD49/51, are able to recruit Drp1 and form the division machinery. In apicomplexan parasites, the function of Fis1 in the recruitment of the division machinery is dispensable, indicating the existence of other essential adaptor proteins. Additionally, in T. gondii, LMF1 seems to bind to Fis1 and might be directly or indirectly involved in the recruitment of the division machinery. (C) Three possible scenarios for mitochondrial and apicoplast fission during schizogony. In the synchronous fission scenario, many fission points will occur simultaneously, resulting in an instant division of the organelle in daughter organelles. In the outside-in fission scenario, the fission points will be formed at the endings of the network-like organelle and daughter organelles will be formed by fission from the endings to the center. In the branching point fission scenario, fission points occur at the branching points of the organelle network, generating smaller fragments.

Mitochondria and plastids both have an endosymbiotic ancestry. It is widely accepted that mitochondria originate from primary endosymbiosis of an ancestral alphaproteobacterium. The apicoplast, being surrounded by four membranes, is the result of a secondary endosymbiotic event. It originates from a red alga that in turn obtained a plastid by endosymbiosis of a cyanobacterium (51). Some early-branching eukaryotes, such as amoebozoa, stramenopiles, and the red alga Cyanidioschyzon merolae, still use a similar division machinery as their bacterial ancestors for fission of their endosymbiotic organelles (62). Bacteria divide by oligomerization of a tubulin-like GTPase, FtsZ, at the cytosolic membrane, corresponding to the matrix side of the inner mitochondrial membrane (IMM), where a so-called Z-ring is formed (Fig. 2A). Together with a dozen other conserved proteins, the Z-ring comprises the divisional machinery and mediates mid-cell constriction (63). We searched for apicomplexan homologs by performing reciprocal blast searches in VEuPathDB and NCBI databases. Confirming previous studies, our bioinformatic analysis did not show any homologs to components of the FtsZ division machinery in Apicomplexa (Table 1), suggesting that these parasites do not harbor this bacteria-like division system (4, 50).

**TABLE 1:** Overview of which endosymbiotic organelle fission proteins are conserved in T. gondii and P. falciparum

Protein(s)	Function(s)	T. gondii homolog(s)	P. falciparum homolog(s)
Drp1, Dnm2 (human), Dnm1 (yeast), Drp3A/B (plant), Drp5B (alga)	Dynamin-related protein, formation of contractile ring	DrpA (TGME49_267800) DrpB (TGME49_321620) DrpC (TGME49_270690)	DYN2 (PF3D7_1037500) DYN1 (PF3D7_1145400) DYN3 (PF3D7_1218500)
hFis1, yFis1	Drp1/Dnm1 adaptor protein	Fis1 (TGME49_263323)	Fis1 (PF3D7_1325600)
Mff/MiD49/MiD51 (human)	Drp1 adaptor protein	NA	NA
Mdv1/Caf4 (yeast)	Dnm1 adaptor protein	NA	NA
FtsZ complex (plants/alga)	Formation of Z-ring	NA	NA
MDR1, PDR1 (plants/alga)	Formation of MD/PD ring	NA	NA
INF2/Spire1C (human)	ER-mediated constriction of the mitochondrion	NA	NA

Many eukaryotes have partially or wholly replaced the ancestral division machinery with a new dynamin-based division machinery (Fig. 2A). Dynamins or dynamin-related proteins (DRPs) are large GTPases that can form ring-like oligomers (dynamin ring) and change conformation to facilitate membrane constriction, scission, or fusion (64). They play a key role in processes such as vesicle budding, cytokinesis, and organelle division. For example, the mammalian dynamin-related protein 1 (Drp1) and yeast dynamin-related GTPase Dnm1 mediate mitochondrial fission.

### Dynamins and their central role in organelle division.

All members of the dynamin superfamily have a similar architecture: a large GTPase domain, a middle domain (MD), and a downstream GTPase effector domain (GED) (64). Three DRPs were identified in T. gondii (*Tg*DrpA to C) and P. falciparum (*Pf*DYN1 to 3) (Table 1). *Tg*DrpA/*Pf*DYN2 and *Tg*DrpB/*Pf*DYN1 have the typical DRP architecture, while *Tg*DrpC/*Pf*DYN3 is apicomplexan-specific and lacks both the GED and MD (65, 66). Surprisingly, *Tg*DrpC, which lacks the two domains that are normally involved in the oligomerization and regulation of the GTPase activity, has recently been indicated to be involved in mitochondrial fission in T. gondii (28). *Tg*DrpC localizes in puncta in the cytoplasm and concentrates at the mitochondrion constriction site during the last steps of cell division, similar to localization of well-studied DRPs in other systems (28, 67–69). Conditional knockdown of *Tg*DrpC showed that this protein is essential for parasite replication and significantly affects morphology of the mitochondrion, apicoplast, IMC, and Golgi (28, 69). Additionally, *Tg*DrpC has been shown to interact with proteins that are homologous to proteins involved in vesicle transport (69). However, expression of a dominant negative form of *Tg*DrpC resulted in impaired mitochondrial segregation and permanent mitochondrial interconnection, suggesting a role in mitochondrial division (28). It is still unclear if *Tg*DrpC actually forms a dynamin ring that mediates mitochondrial constriction or if it plays a more indirect role in mitochondrial fission. Although the P. falciparum ortholog *Pf*DYN3 is predicted to be essential (70), expression data do not unanimously support a role in mature asexual blood stages but appear rather variable across different studies (https://plasmodb.org). Thus, it still remains to be determined if *Pf*DYN3 plays a role in mitochondrial fission or has additional functions.

*Tg*DrpB is thought to be involved in the biogenesis of secretory organelles in T. gondii. Conditional ablation of *Tg*DrpB resulted in the parasites that lack micronemes and rhoptries and were unable to escape or invade the host cells (66). *Tg*DrpB is possibly involved in formation of vesicles for the secretory pathway that form the secretory organelles. The P. falciparum homolog *Pf*DYN1 is essential for parasite survival and is suggested to play a role in vesicle budding during hemoglobin uptake (71–73).

In plants and algae, plastid division relies on the combined action of the ancestral Z-ring and the eukaryotic dynamin ring, and it is structurally and functionally highly similar to the mitochondrial division machinery (74). A similar division machinery is used by some heterokonts that acquired their plastids via secondary endosymbiosis and therefore harbor four plastid membranes (75). However, apicomplexan parasites lack homology to both the FtsZ division system and the plant and alga dynamin division system (ARC5 and Dnm2, respectively). This suggests that Apicomplexa have lost the primary chloroplast division machinery and developed a new mechanism for the division of the apicoplast that is different from the division machinery of previously studied plastids. (25, 76).

Phylogenetic analysis has shown that *Tg*DrpA and *Pf*DYN2 are distinct from chloroplast division proteins and cluster together with other DRPs, such as human Drp1, that are involved in fission of the outer mitochondrial membrane (OMM) (76). Surprisingly, van Dooren et al. have shown that *Tg*DrpA is involved in apicoplast fission in T. gondii (25). Overexpression of a nonfunctional *Tg*DrpA resulted in severe growth defects of the parasite and impaired apicoplast segregation. Additionally, *Tg*DrpA localizes to the apicoplast fission point during endodyogeny, where a potential dynamin ring can be expected (25). This would mean that the apicoplast uses a unique plastid division machinery that is highly similar to the mitochondrial dynamin-based division machinery. Although *Pf*DYN2 has been shown to have GTPase activity *in vitro*, it remains to be determined if it has a role in apicoplast division in P. falciparum (76). An interesting observation entered as a comment by Ellen Yeh in PlasmoDB (http://plasmodb.org) lends support for roles beyond apicoplast fission. She noted that, while knockdown of this protein results in growth inhibition, this inhibition was not rescued by isopentenyl pyrophosphate as would be expected when *Pf*DYN2 would function at the apicoplast exclusively.

In addition to the FtsZ and dynamin rings, electron microscopy studies have identified another electron-dense specialized ring structure at the division site of plastids and mitochondria in numerous photosynthetic eukaryotes (77–79). The plastid division (PD) ring comprises two or three types of specialized electron-dense ring structures: (i) the outer PD ring, which forms the main skeletal structure of the plastid division machinery and consists of a ring-shaped bundle of nanofilaments on the cytosolic side of the organelle membrane (79), (ii) the inner PD ring, which is formed on the inside of the inner plastid membrane, and (iii) an intermediate PD ring, which has been observed in the intermembrane space of C. merolae and the green alga Nannochloris bacillaris (80, 81). Although the conservation of the middle PD ring is less clear, the outer and inner PD rings have been found in many members of the plant kingdom and have been observed at division sites of multiple types of plastids, including proplastids, amyloplasts, and chloroplasts (82). In some lineages of heterokonts, which harbor a four-membrane plastid of secondary endosymbiosis, an outer PD ring has been observed (83, 84). However, it remains unclear if other secondary endosymbiotic plastids, including the apicoplast, also harbor this PD ring for their organelle division. Interestingly, in lower eukaryotes a counterpart of the PD ring was found in mitochondrial division (79, 85). This mitochondrial division (MD) ring also consists of an inner MD ring located at the matrix side of the IMM and an outer MD ring at the cytosolic side of the OMM. In contrast to the PD ring, MD rings have so far been identified only in early-branching eukaryotes, although some studies in yeast and human cells also identified electron-dense structures at the mitochondrial division site (86, 87).

Both the PD and MD rings have been shown to consist of polyglucan filaments that form a belt-like structure (88, 89). Plastid-dividing ring 1 (PDR1) is a glycosyltransferase protein in *C. merolae* that is embedded in the polyglucan filaments of the outer PD ring at the plastid division site and is thought to play an important role in the elongation of the glucan chain. The recently identified mitochondrial analogue mitochondrion-dividing ring 1 (MDR1) has little sequence similarity with PDR1 (89). However, MDR1 and PDR1 both harbor a glycosyltransferase domain that belongs to the type-8 subgroup of the glycosyltransferase family, and they have homologous functions in plastid and mitochondrial division. PDR1 orthologues have been identified in other land plants, but it remains unclear if it is conserved in other eukaryotes or apicomplexan parasites. Further studies are needed to investigate if apicomplexan parasites harbor a PD ring in their apicoplast division machinery.

### Adaptor proteins, recruiters of the organelle division machinery.

DRPs are recruited to the site of fission by adaptor proteins that associate with the organelle membrane (Fig. 2A) (90, 91). After recruitment of DRPs, the multimeric DRP structures are assembled and the dynamin ring is formed. Although DRPs are well conserved, adaptor proteins are highly variable between different eukaryotes and are not related by primary amino acid sequence, predicted secondary structure, or domain composition (90). Several mitochondrial adaptor proteins have been identified in human (Mff, MiD49, MiD51, Fis1) and yeast (Mdv1, Caf4, Fis1) (Table 1). Interestingly, the membrane-anchored Fis1 is the only mitochondrial adaptor protein that is highly conserved among eukaryotes that contain mitochondria. In yeast, Fis1 is the only known membrane-bound adaptor protein and is essential for the membrane recruitment of the other fission proteins Mdv1 and Caf4, which in turn recruit the mitochondrial fission machinery (Fig. 2B) (92, 93). Conversely, there is redundancy in the role of human Fis1 where Drp1 recruitment can be facilitated by the other adaptor proteins, Mff, MiD49, and MiD51 (Fig. 2B) (94, 95). Overexpression of human Fis1 leads to mitochondrial fragmentation, indicating a role in mitochondrial dynamics (96). Although T. gondii and P. falciparum lack homologs to other mitochondrial adaptor proteins, both parasites harbor a Fis1 ortholog (Table 1) (28, 97). Fis1 is a relatively small protein of approximately 16 kDa and contains two tetratricopeptide domains, a C-terminal transmembrane domain and a small C-terminal tail (97, 98). N-terminal tagging of Fis1 in T. gondii and P. falciparum confirmed its mitochondrial localization, which depends on its C-terminal transmembrane domain and the C-terminal tail. Conditional knockdown or knockout of Fis1 in T. gondii and P. falciparum did not result in a growth defect or affect mitochondrial morphology (28, 97). This suggests that Fis1 is dispensable and does not play an essential role in mitochondrial fission in these parasites. However, T. gondii parasites lacking Fis1 were less susceptible to the polyether ionophore monensin, which induces morphological changes of the mitochondrion as a result of constrictions in the OMM (98). Additionally, mislocalization of Fis1 to the cytoplasm by the truncation of the C-terminal transmembrane domain in T. gondii caused significant alterations in mitochondrial morphology. These results indicate a role for Fis1 in mitochondrial morphology and suggest that Fis1 might interact with other proteins that are critical for mitochondrial morphology which are pulled away from their action site upon Fis1 mislocalization. Jacobs et al. identified a novel OMM protein interacting with Fis1 in T. gondii, which they named the lasso maintenance factor 1 (LMF1). *LMF1*-disrupted parasites show significant growth defect, altered mitochondrial morphology, and failure of proper mitochondrial segregation during endodyogeny (98). LMF1 might be localized to the mitochondrion by protein-protein interaction with Fis1, where it might be directly or indirectly involved in the recruitment of the fission machinery. Additionally, knockout of *LMF1* resulted in sperm-like and collapsed mitochondrial morphologies, which could be due to the loss of contact sites between the mitochondrion and the IMC. However, further research is needed to confirm these roles of LMF1 in mitochondrial morphology and division. We were not able to identify an *LMF1* ortholog in P. falciparum.

It is clear that our understanding of the proteins and mechanisms involved in mitochondrial fission in apicomplexan parasites is very limited. As the role of Fis1 is dispensable, it is likely that there are other, as yet unidentified adaptor proteins in apicomplexan parasites that are essential for the recruitment of the division machinery.

## CONCLUSIONS AND PERSPECTIVES

Apicomplexan parasites have two different endosymbiotic organelles but harbor only one of each. The mitochondrion and apicoplast are both essential for parasite development. This makes proper division and distribution over daughter cells essential. Parasites utilize fission machineries to divide their mitochondrion and apicoplast that have highly diverged from their endosymbiotic ancestors and their human host. Therefore, these might form an attractive target for drug development. While some progress has been made toward a better understanding of the molecular processes involved, most of the fundamental mechanisms underlying organelle division remain elusive.

Morphological studies in both T. gondii and P. falciparum revealed that apicoplast division precedes mitochondrial division, which happens only during the final stages of cell division. In contrast to T. gondii that needs to divide and distribute the organelles over two daughter cells, P. falciparum must divide its mitochondrion and apicoplast in up to 32 fragments during blood-stage schizogony and even in thousands during sporozoite and liver-stage merozoite formation. Here, we propose three possible scenarios for division of the mitochondrion and apicoplast in P. falciparum (Fig. 2C).

(i) Synchronous fission: instant division of the organelle into daughter organelles with many simultaneous fission points at the organelle.

(ii) Outside-in fission: organelle division takes place at the ends of the network-like organelle, which are split off until the whole organelle is divided into daughter organelles.

(iii) Branching point fission: branching points of the mitochondrial network are the initial fission sites generating a few smaller fragments, which are then divided until all the daughter organelles are formed.

The ability to visualize and manipulate organelles and sub-organellar structures in high resolution in a noninvasive manner is critical for understanding which, if any, of these scenarios apply to organelle fission in P. falciparum. Technological developments, such as lattice light sheet microscopy and high-resolution live imaging, together with the development of noninvasive organelle markers will enable the capturing of the process of organelle division in apicomplexan parasites in four dimensions.

In conclusion, the components and mechanisms of the organelle division machinery in apicomplexan parasites remain largely unknown. The endosymbiotic organelle division machinery in eukaryotes includes at least one contractile ring, FtsZ- or dynamin-based, that mediates mid-organelle constriction. Although apicomplexan parasites lack components of the ancestral FtsZ-based division machinery, they do harbor three DRPs, of which two have been indicated to be involved in apicoplast or mitochondrial division. Further studies are needed to verify the functions of these proteins in organelle division. In contrast to the DRPs, adaptor proteins that recruit the division machinery are highly variable in eukaryotes. T. gondii and P. falciparum both harbor a Fis1 homolog, which is a highly conserved and extensively studied adaptor protein in humans, yeast, plants, and algae. Although its function in mitochondrial fission in these parasites needs to be verified, dispensability of this protein indicates that there are unidentified and more important adaptor proteins that are central to recruitment of the division machinery. Despite a gradually expanding experimental genetics toolbox, ever better imaging resolution, e.g., the successful implementation of expansion microscopy (99) and focussed ion beam scanning electron microscopy (3), and novel proteomics-based approaches to study protein-protein interaction and protein complexes (10, 100), the studying of such short-lived interactions will remain a significant challenge. Nevertheless, the search for the apicomplexan endosymbiotic organelle division machinery or machineries continues. Understanding the molecular basis of the organelle division machinery in apicomplexan parasites will enable a better understanding of this fascinating and essential process.
